# Distribution and Enrichment Regularity of Trace Elements in Meitan Cuiya Tea and Soil

**DOI:** 10.3390/toxics13090741

**Published:** 2025-08-31

**Authors:** Jia Wei, Haiyun Zhou, Qiao Liu, Lin Bai, Minjie Han, Gendi Liu, Shuyan Pei, Fumei Zhang, Xiaojing Tian, Guoheng Zhang

**Affiliations:** 1 School of Life Sciences and Engineering, Northwest Minzu University, Lanzhou 730030, China; weijiapku@xbmu.edu.cn (J.W.); y230830456@stu.xbmu.edu.cn (H.Z.);; 2 Gansu Tech Innovation Center of Animal Cell, Biomedical Research Center, Northwest Minzu University, Lanzhou 730030, China; 3 College of Basic Medicine, Northwest Minzu University, Lanzhou 730030, China; 4 Gansu Engineering Research Center of Ecological Environment Intelligent Networking, College of Electrical Engineering, Northwest Minzu University, Lanzhou 730030, China

**Keywords:** Meitan tea, soil, soil–tea system, trace elements, bioconcentration factor

## Abstract

**Purpose:** This study aimed to investigate the migration and distribution characteristics of trace elements in the soil–tea system in the Cuiya tea area of Meitan County, Guizhou Province. **Methods:** The contents of trace elements (Cd, Fe, La, Mg, Mn, Ni, Se, Pr, Sm, Zn) in tea and soil samples were determined by inductively coupled plasma emission spectrometry (ICP-OES). **Results:** The average contents of heavy metals in soil and tea from Meitan County were below the Chinese national standards, while also meeting the criteria for selenium enrichment. Within the soil–tea system, Mn in tea leaves exhibited a significant negative correlation with soil Mn, while Cd showed a significant positive correlation with soil Cd. This pattern was consistent across both the topsoil and subsoil. The tea plants exhibited a high enrichment capacity for Mn, Mg, and Zn, but a low capacity for Sm, Fe, and Cd. Among the studied areas, the enrichment effect was most pronounced in SL, XH, and MJ towns. **Conclusions:** Significant spatial variations were observed in the concentrations of trace elements in both tea and soil across the Meitan tea area. This study provides a scientific basis for understanding the enrichment and migration of trace elements within the soil–tea system of Meitan County, Guizhou, and for tracing the geographical origin of its tea.

## 1. Introduction

China, recognized as the birthplace of tea culture, is also the largest tea-producing, -exporting, and -consuming country [1,2]. The southwestern region of China represents the country’s most significant tea-growing area. Within this region, Guizhou Province ranks as China’s second largest premium tea production base [3]. Notably, as Guizhou’s foremost tea-growing region, Meitan County is an ideal location for tea cultivation and production, owing to its unique natural resources, favorable climate, and distinctive topography [4,5]. However, in recent years, anthropogenic influences, such as industrial waste discharge, traffic emissions, and sewage irrigation, as well as pesticide and fertilizer application, have caused serious pollution in the soil, thereby affecting the content of trace elements in tea soil [6,7]. Soil serves as the essential growth medium for tea, and its nutrient element content is crucial for both plant growth and tea quality [8]. Cu deficiency in tea plants will inhibit root growth [9], Zn deficiency can easily lead to leaf abscission [10], and Fe deficiency can cause symptoms such as leaf chlorosis [11]. However, excessive accumulation of trace elements in tea soils causes environmental contamination and increases the uptake of these elements by tea plants [12]. Thus, an increase in trace element uptake by tea crops may not only affect the quality of tea leaves but also threaten public health.

The soil–tea system is a comprehensive ecological and agricultural concept, emphasizing the interaction between the soil environment and tea plant growth and tea quality [13]. A health risk assessment of heavy metals in the soil-crop system of central southwest Guizhou revealed that the consumption of corn and tea by local residents posed a low health risk [14]. The main methods for evaluating soil cleanliness in tea areas include the single pollution index (*P_i_*), the composite index method, the index of geoaccumulation (*I_geo_*), and the enrichment coefficient method [15]. All of these methods have been proven scientifically effective. The metal pollution in the Suape estuarine system, Brazil, was evaluated using enrichment factors and the *I_geo_*. The results indicated moderate contamination of Zn, Ga, V, and Pb, primarily due to anthropogenic influences [16]. In addition, the accumulation, spatial enrichment, and ecological risk of heavy metals in agricultural soil samples in Dhaka District were evaluated by enrichment factor, *I_geo_* and *P_i_*. It was found that more than 90% of the soil samples in this area were polluted by high levels of Cr and Cd, and about 73% of the soil samples were moderately polluted by heavy metals [17]. However, previous studies ignored the characteristics and soil-to-plant migration of trace elements in tea plantations, along with the relationships between: (a) trace elements in soils and tea leaves, and (b) soil properties and trace elements in both soils and leaves.

This study aims to investigate the migration of trace elements and their characteristics in the soil–tea system in the Meitan Cuiya tea area of Meitan County. Ten trace elements (Mg, Zn, Se, Cd, Fe, Mn, Ni, La, Pr, and Sm) were determined simultaneously by using an inductively coupled plasma optical emission spectrometer (ICP-OES) in tea and soil systems in 12 different Meitan Cuiya tea areas. The specific objectives of this study are as follows: (1) to determine the contents of heavy metal elements in the tea and soil systems of 12 different tea plantations in Meitan County, Guizhou Province, using ICP-OES, and to perform a correlation analysis; (2) to evaluate the pollution levels in tea and soil using the *P_i_* and *I_geo_* indices; and (3) to characterize the enrichment patterns of trace elements in the soil–tea system via the bioconcentration factor (BCF).

## 2. Materials and Methods

### 2.1. Samples and Chemical Reagents

A multi-element standard stock solution (1000 μg·mL^−1^), containing Cd, Fe, La, Mg, Mn, Ni, Se, Pr, Sm, and Zn, was purchased from Beijing Inspection and Certification Co., Ltd. (Beijing, China). Nitric acid (GR), hydrochloric acid (GR), and perchloric acid (GR) were purchased from Yantai Shuangshuang Chemical Co., Ltd. (Yantai, China).

### 2.2. Sample Preparation

**Figure 1:**
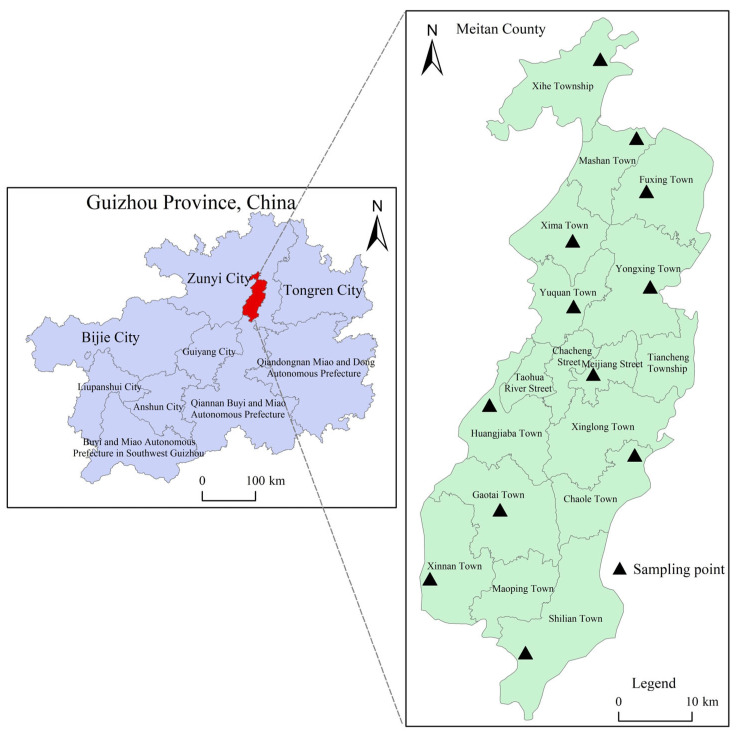
Geographical distribution of sampling localities in Meitan County of Guizhou. Note: The red part in the left represents Meitan County of Guizhou Province; the triangles in the right designate the sampling points.

According to the overall distribution of tea areas and topographic features of Meitan County, Guizhou Province, 12 representative towns were selected for the collection of Meitan tea samples (Meitan Cuiya); the collection of Meitan tea samples was completed in autumn (early September 2020), and a total of 24 soil samples corresponding to the topsoil of the Meitan tea area (with the removal of surface organic residues and sampling depths ranging from 0 to 20 cm) and the subsoil (from 20 to 40 cm) were collected. The final tea leaves, topsoil, and subsoil samples were each weighed to 500 g. The geographical locations of sampling points were shown in Figure 1, and the de-tailed sample information were shown in Table 1.

Soil sample preparation: Each soil sample (>500 g) was manually cleaned to remove foreign matter, mixed thoroughly and then reduced to 100 g by the quadratic method. Soil samples were dried to constant weight at 40 °C in a DHG-9146 A drying oven (Shanghai Jinghong Experimental Equipment Co., Ltd., Shanghai, China) and sieved through a 60-mesh sieve. Soil samples for trace element analysis were digested with aqua regia (nitric acid: hydrochloric acid = 1:3)- perchloric acid mixture [18]. The digestion was performed at 100 °C for 2 h in a muffle furnace (SX2-4-10, Shanghai Xin Yi Instrument and Meters Co., Ltd., Shanghai, China). Each sample was prepared in quintuplicate.

Tea sample preparation: Tea leaves were first rinsed with ultrapure water (18.2 MΩ cm) for 10–15 s, then dried to a constant weight in a DHG-9146 A drying oven (Shanghai Jinghong Experimental Equipment Co., Ltd., Shanghai, China) at 40 °C. Subsequently, the dried samples were finely ground and ashed at 600 °C for 4 h in a muffle furnace (SX2-4-10, Shanghai Xin Yi Instrument And Meters Co., Ltd., Shanghai, China). The ash was dissolved in 5 mL diluted nitric acid (5% HNO3) and filtered through a 0.22 μm water filter membrane.

### 2.3. Methods

#### 2.3.1. Operating Parameters of ICP-OES

The elemental analysis was performed using an Agilent 5110 inductively coupled plasma optical emission spectrometer (Agilent Technologies Ltd., Beijing, China) with the following optimized operating conditions: radio frequency power of 1200 W; the plasma gas flow rate was 12 L·min^−1^; the auxiliary gas flow rate was 1 L·min^−1^; the observation method was SVDV; the observation height was 8.

#### 2.3.2. Standard Curve Protracting of Elements

In this study, parallel and standard soil samples (GBW07403) were used for soil quality control, and parallel and standard tea samples (GBW07605) were used for tea quality monitoring. The average recovery of heavy metals (Cd, Fe, La, Mg, Mn, Ni, Se, Pr, Sm, Zn) in standard samples was 90~105%. In addition, as shown in Table 2, the correlation coefficient r value of each element standard curve was between 0.9908 and 0.9999. The standard curve showed good linearity in the specified concentration range, and the relative standard deviation (RSD) values of each element were between 0.37% and 3.68%, all less than 4%. Therefore, the established method has good repeatability.

#### 2.3.3. *P_i_* Evaluation

The *P_i_* Evaluation [19], was employed for soil quality assessment, following the National Soil Pollution Evaluation Technical Regulations (Measures for the administration of environmental monitoring [2008] No. 39). It is the basis for environmental classification, environmental quality index and comprehensive evaluation. The calculation formula of the *P_i_* is as follows:

(1) $$P_{i}=C_{i}•S_{i}^{-1}$$

where *P_i_* is the *P_i_* of the pollutant *i* in the soil, *i* represents a certain pollutant, *C_i_* is the measured value of the pollutant *i* in the soil (mg·kg^−1^), and *S_i_* is the background value of the pollutant in the soil (mg·kg^−1^).

For the evaluation standard, when *P_i_* ≤ 1, the soil is free of pollution. When *P_i_* > 1, the heavy metals in the soil exceed the standard, which impact on the growth and development of crops.

#### 2.3.4. *I_geo_* Evaluation

With the background value of Guizhou A layer soil as reference standard, the degree of heavy metal (with relative density more than 4.5 g/m^3^) pollution of 12 towns of Meitan County was evaluated by index of *I_geo_.* The results were compared with *I_geo_* Classification criteria (Table 3) [20]. The *I_geo_* was calculated:

(2) $$I_{\mathrm{geo}}={log}_{2}[C_{n}•{\left(1.5B_{n}\right)}^{-1}]$$

where *C_n_* is the concentration of element *n* in the sample soil, *B_n_* is the background concentration, and 1.5 is the correction index, which is usually used to characterize sedimentary characteristics, rock geology and other influences.

#### 2.3.5. pH Determination

Soil pH was detected according to the “Agricultural Standard Determination of Soil pH NY/T 1377-2007” [21]. The soil and water were mixed at a ratio of 1:2.5 (*w*/*v*), stirred for 2 min, and stood for 30 min. The pH was determined by PHS-3 C acidity meter (Shanghai Instrument Electrical Science Instrument Co., Ltd., Shanghai, China).

#### 2.3.6. Bioconcentration Factor for Trace Element

In order to explore the absorption of elements in tea soil, bioconcentration factor (BCF) [22] was employed to evaluate trace element transfer from soil to tea plants. The higher the BCF value, the greater elemental accumulation capacity in tea plants. The bioconcentration factor calculation formula is as follows:

(3) $$BCF=C_{\mathrm{tea}}/C_{\mathrm{soil}}$$

Among them, C_tea_ and C_soil_ were the concentrations of trace elements in tea and the corresponding soil (mg·kg^−1^), respectively, measured by dry weight.

### 2.4. Statistical Analysis

The experimental data were processed by Microsoft Excel 2010, SAS 9.4 and SPSS 21 software (*p*-values less than 0.05 were considered statistically significant), one-way analysis of variance was performed on the topsoil and subsoil (Table 4) and tea (Table 5) in 12 townships in Meitan County. Pearson analysis was performed on the correlation heat map. and the figure was drawn by ArcGIS 10.2, Origin 8.0, and TB-tools 1.046.

## 3. Results

### 3.1. Characteristics of Element Content of Soil

#### 3.1.1. Soil Element Content

The concentrations and statistical analysis of 10 elements (Cd, Fe, La, Mg, Mn, Ni, Se, Pr, Sm, and Zn) in the tea soil of 12 towns in Meitan County are summarized in Table 4. The average total concentrations of these elements in the topsoil of Meitan Couty were 0.767, 3835.8, 1.44, 64.42, 0.565, 3.991, 5.07, 1.11, 1.089, and 1.402 mg·kg^−1^, respectively. In contrast, the concentrations in the subsoil were 0.79, 3822.5, 1.45, 70.6, 0.53, 4.23, 5.1, 1.93, 1.09, and 1.31, respectively. In the topsoil, the concentration of La varied significantly across the 12 towns. Similarly, the concentration of Cd also showed significant variation, except in XH and MJ towns. Moreover, Fe concentration exhibited significant differences in all towns except XN and YX. Mg concentration varied significantly except in MJ and XN, while Mn showed significant variation across all towns except XH and FX. For the subsoil, the concentration of La differed significantly across all towns except GT and HJ. Similarly, Ni concentration showed significant variation except in XH and SL, while Zn differed significantly in all towns except YQ and YX.

Notably, the concentration of Fe and Mg were markedly higher than those of the other elements in topsoil and subsoil. The selenium concentration in the topsoil and subsoil was 5.07 mg·kg^−1^ and 5.10 mg·kg^−1^, respectively, complying with the DB41/T 1871-2019 standard for selenium-rich soil requirements [23]. Compared with GB 15618-2018 [24], the heavy metals Ni (<60 mg·kg^−1^) and Zn (<200 mg·kg^−1^) in the topsoil and subsoil did not exceed the regulatory limits. Although the heavy metal Cd (0.3 mg·kg^−1^ < Cd < 1.5 mg·kg^−1^) exceeds the green risk value specified for soil, it remained below the maximum value for agricultural soil pollution risk control.

#### 3.1.2. Evaluation of Indicators for Heavy Metal Pollution in Soil

**Figure 2:**
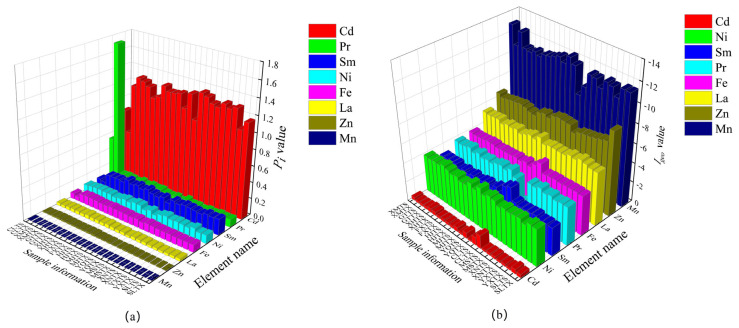
Soil *P_i_* value (**a**) and *I_geo_* value (**b**) in Meitan tea area.

In order to evaluate the heavy metal pollution status in the Meitan tea area, *I_geo_* and *P_i_* for each trace element were analyzed. The results showed that the average *I_geo_* value of trace elements in Meitan area was below 0, and the average *P_i_* value was below 1 except in the townships of SL and CL (Figure 2). Therefore, the soil in Meitan was not contaminated by the elements of Mn, Zn, La, Fe, Ni, and Sm, indicating that the overall soil quality is relatively clean.

On the other hand, among the elements studied, Mn exhibited the most negative *I_geo_* values (farthest from 0) and the lowest *P_i_* values (farthest from 1) in the Meitan tea areas, indicating no contamination. In contrast, the *I_geo_* values of Cd were close to 0, and its *P_i_* values were generally greater than 1 (except in CL township), suggesting a potential risk of Cd contamination. Furthermore, in the subsoil of CL township, Pr showed *P_i_* values greater than 1, despite having *I_geo_* values below 0. These results indicate that the pollution levels of Cd and Pr in the Meitan tea areas were close to critical thresholds. It is, therefore, recommended to mitigate the input of toxic substances containing Cd and Pr in these areas. This proactive measure is essential to prevent future elemental pollution, maintain soil cleanliness, and ensure a healthy environment for plant growth.

#### 3.1.3. Correlation Analysis of Soil Element Content and pH

**Figure 3:**
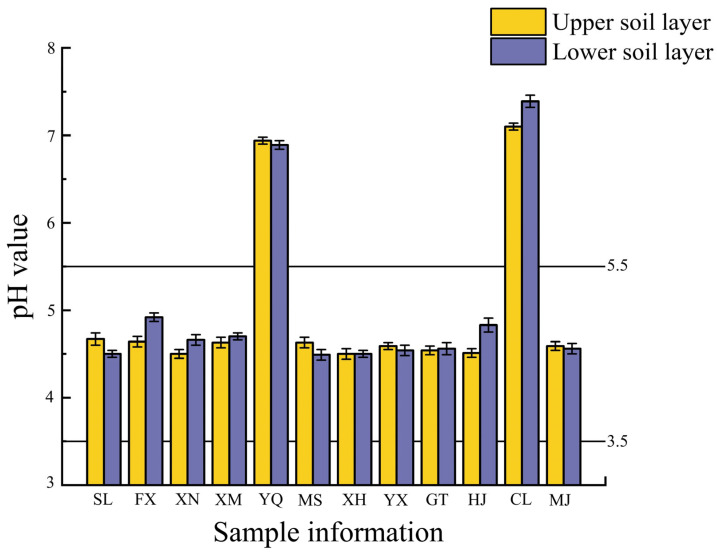
Columnar diagram of pH of topsoil and subsoil in 12 towns of Meitan County.

The stability of soil pH is crucial for ensuring the yield and quality of tea [25], the optimum soil pH for tea tree growth ranges from 3.5~5.5. As shown in Figure 3, the soil pH for 12 towns in Meitan County, ranged from 4.49 ± 0.06 to 7.39 ± 0.07. From the perspective of soil pH, the soil in SL, FX, XN, XM, MS, XH, YX, TG, HJ, and MJ townships was more suitable for tea plant growth. In contrast, the soil in YQ and CL townships had pH levels exceeding 5.5. Therefore, the application of physiological acidic fertilizers, such as ammonium sulfate and ammonium chloride is recommended for promotion of tea growth.

**Figure 4:**
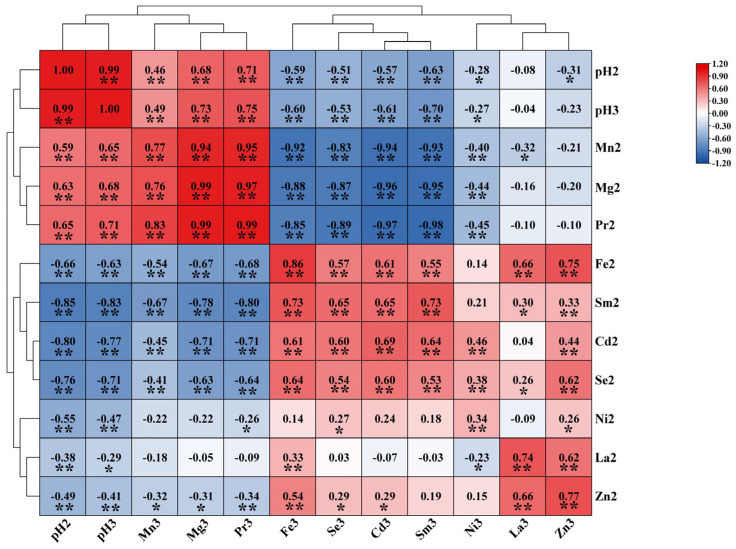
Heat map of the correlation between topsoil, subsoil, and pH (2: topsoil; 3: subsoil). * Represents correlation at the 0.05 level; ** represents correlation at the 0.01 level.

To examine the correlation between soil element content and pH, we performed a correlation analysis. The resulting heatmap is presented in Figure 4. In the topsoil of the study area, pH value was significantly positively correlated with the content of Mg, Mn and Pr, while significantly negatively correlated with the content of Cd, Fe, La, Ni, Se, Sm and Zn. The correlation between trace elements and pH value in the subsoil was the same as that in the topsoil. Furthermore, a highly significant positive correlation between pH of the topsoil and subsoil was observed in the study area.

### 3.2. Characteristics of Element Content in Tea

#### 3.2.1. Tea Element Content

The content of 10 elements in tea and the result of variance analysis (*p* < 0.05) for 12 tea areas in Meitan County, Guizhou are shown in Table 5. There were significant differences in Fe content among towns except for GT and CL. According to NY/T 659-2003 [26], the average content of Cd in tea in Meitan area was below the Chinese national standard of 1mg·kg^−1^. Additionally, according to GH/T 1090-2014 [27], the tea leaves from XN, XH, and MJ towns met the selenium-rich tea standard, with selenium content exceeding 0.2 mg·kg^−1^. The tea leaves of the other 7 towns in Meitan County also contained selenium, with the concentrations ranging from 0.093 ± 0.013 to 0.171 ± 0.027 (mg·kg^−1^). The above results indicate that the overall quality of tea in the tea-growing areas of Meitan County is relatively good.

#### 3.2.2. Correlation Analysis of Tea Trace Element Content, Soil Trace Element Content and Soil pH

**Figure 5:**
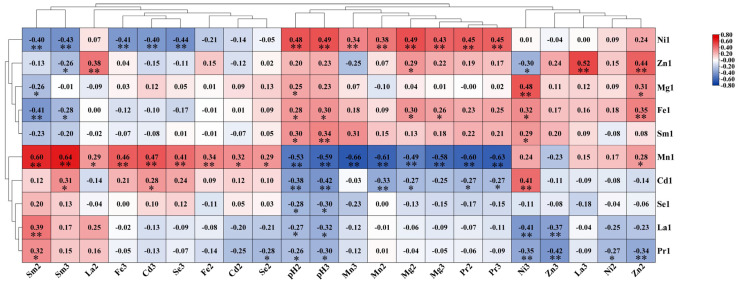
Tea–soil–pH correlation heat map (1: tea, 2: topsoil, 3: subsoil). * Represents correlation at the 0.05 level; ** represents correlation at the 0.01 level.

Correlation analysis was carried out to examine the relationships between the element contents of tea and both the topsoil and subsoil, as well as the relationships between the element content of tea and the soil pH. The resulting correlation heat map is shown in Figure 5. As shown in Figure 5, Mn in tea leaves had strong negative correlation with Mn, Mg and Pr in both topsoil and subsoil. Conversely, Mn in tea leaves exhibited strong positive relationship with Sm, Fe, Cd and Se in the soil. Zn and Cd in tea leaves showed significant positive correlations (*p* < 0.05) with those in both topsoil and subsoil. Additionally, La in tea leaves exhibited significant positive relationship with La in topsoil. Moreover, strong negative correlations were identified between the soil pH (in both topsoil and subsoil) and the content of Cd, Mn, La, Se, and Pr in tea leaves. In contrast, significant positive relationships were found between soil pH and the content of Ni, Sm, and Fe elements in tea leaves (*p* < 0.05).

**Figure 6:**
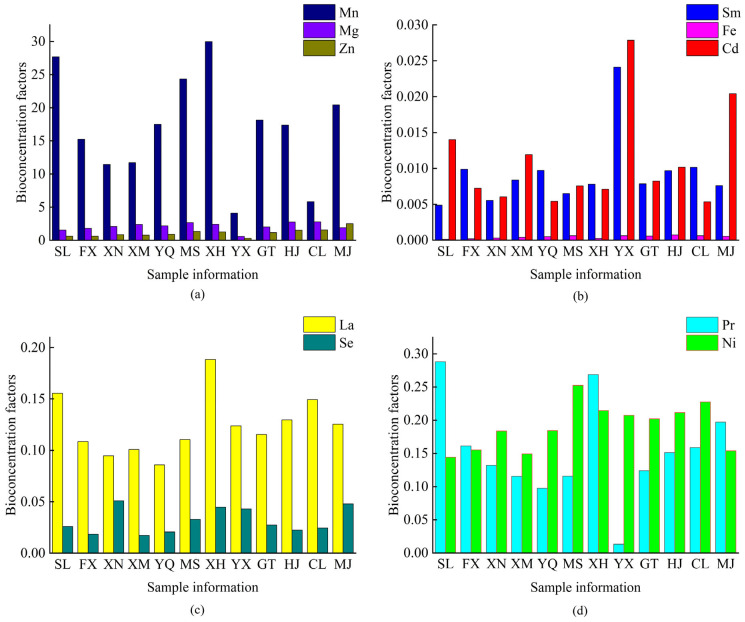
The element enrichment ability of Meitan tea tree. The enrichment ability of Mn, Mg and Zn (**a**), the enrichment ability of Sm, Fe and Cd (**b**), the enrichment ability of La and Se (**c**), the enrichment ability of Pr and Ni (**d**).

To understand the trace element enrichment characteristics in Meitan tea leaves, BCF were calculated for each trace element. The BCF of Cd, Fe, La, Mg, Mn, Ni, Se, Pr, Sm, and Zn in soil–tea system across the 12 towns of the Meitan tea area is shown in Figure 6. There were great differences in the enrichment capacities of tea trees for the 10 elements in the soil among different towns. Among them, the BCF values for Mn were significantly higher than those for the other elements, with the BCF value for Mn in XH town reaching as high as 30. The BCF values of Mn element across different towns are ranked as follows: XH > SL > MS > MJ > GT > YQ > HJ > FX > XM > XN > CL > YX. In contrast, The BCF values of Fe were notably lower compared to those of other elements.

## 4. Discussion

Soil elements are closely related to the growth and quality of tea plants. Different elements directly or indirectly affect the physiological metabolism, yield and chemical composition of tea plants [28]. Significant differences were found in the soil La content among various tea-producing areas in Meitan. Furthermore, the soil contents of Fe and Mg in these areas were generally high. As one of the rare earth elements, La element in soil has a certain regulatory effect on tea plant growth and tea quality at low concentration, which is mainly reflected in enhancing plant photosynthesis [29]. In addition, Fe and Mg elements are the core components of chlorophyll synthesis. Furthermore, both the topsoil and subsoil in Meitan area met the standard of selenium-enriched tea. This finding is consistent with a study on the accumulation and health risk assessment of heavy metal (loid) in soil-crop systems in Central Guizhou, Southwest China [14]. On the other hand, there were differences in the content of tea elements in Meitan area, especially Fe, Mg and Mn. These variations might arise from differences in the growth environment and soil conditions. Using ICP-AES, the trace element content in tea soils from Guizhou Province, China, were analyzed, showing that the soil element content varied by region, and that the enrichment capacity of tea plants differed accordingly [30]. This is consistent with the results of this study.

Soil pH is considered as a vital factor influencing the transformation of trace elements from soil to plants [31]. Increased soil acidity may elevate the solubility of trace elements in the soil, and consequently increasing the uptake of the elements by plants [32]. Several Studies have reported negative correlations between soil pH and translocation of trace elements to plants [33]. For instance, an investigation into how pH and organic matter in soil affect heavy metal uptake by rice, found a significant negative correlation between soil pH and Cr, Cu, Fe, Mn, Pb or Zn content in both paddy soil and rice straw [28]. Consistently, strong negative correlations were found between the soil pH and the content of Cd, Mn, La, Se, and Pr in tea leaves in Meitan area. In contrast, Ni, Sm, and Fe in tea leaves were found positively correlated with soil pH.

The trace elements of the roots of the tea plants were not concerned in our study, and the transfer of trace elements in the tea plants from root to shoot might be complicated. It is worth noting that among the 10 trace elements studied, Mn has the strongest enrichment ability in tea and Fe is the weakest, which was consistent with the prior reports about accumulation of heavy metals in tea leaves [13]. Mn is an essential trace element for plants, which is involved in key physiological processes such as photosynthesis, enzyme activation and nitrogen metabolism [34]. As a perennial evergreen plant, tea plant has active metabolism and high demand for Mn [35]. Tea plants are suitable for growing in acidic soil with pH 4.5–6.0, and acidic conditions will promote the dissolution of Mn in soil, improve its bioavailability, and facilitate root absorption [36]. This finding is consistent with our results, which revealed a significant negative correlation between soil pH and Mn content in tea leaves. Not only the acidity of the soil but also the accumulation of Sm, La, Fe, Cd in soil might promote the enrichment of Mn in tea leaves, as indicated by our correlation study. In this study, Mn levels in tea leaves exhibited a negative correlation with soil Mn content, indicating easy absorption and translocation of Mn by tea trees. The efficient absorption and translocation of Mn can be attributed to the corresponding gene expression with in tea plants [34]. Furthermore, the stability of existing forms of Mn in tea leaves might also contribute to its accumulation [13]. Additionally, positive correlations were found between the concentrations of Zn, Cd, and La in tea leaves and their respective levels in the soil, indicating that the accumulation of these elements in tea is primarily determined by their availability in the soil.

## 5. Conclusions

In this study, the contents of trace elements (Cd, Fe, La, Mg, Mn, Ni, Se, Pr, Sm, Zn) in soil and tea samples from the Meitan tea area were determined. The results showed that the contents of Fe and Mg in the soil of the Meitan tea area were high. Although the topsoil and subsoil of the Meitan tea area met the standard of selenium-enriched tea, the *Pi* values of Cd and Pr elements in this area were close to 1. Therefore, it is recommended that emissions of Cd and Pr in the studied tea area be strictly controlled to prevent soil contamination and ensure the production of safe tea. In addition, the content of Mn in tea leaves was significantly negatively correlated with the Mn element in both the topsoil and subsoil, and the BCF of Mn element in Meitan tea was the highest, indicating that Mn element was easy to accumulate in tea. These findings provide a foundational reference for understanding the characteristics of trace elements in the soil–tea system in Meitan, Guizhou, China.

## Figures and Tables

**Table 1:** Information of sampling points of 12 towns in Meitan County.

**Serial** **Number**	**Sampling Point**	**Number of Samples**	**Latitude and Longitude**	**Tea**	**Soil Samples**	
					**Topsoil** **(0–20 cm)**	**Subsoil** **(20–40 cm)**
1	Shilian town	5	27°25′42.77″ N, 107°25′3.79″ E	SLC	SLU	SLL
2	Fuxing town	5	27°59′48.16″ N, 107°37′18.15″ E	FXC	FXU	FXL
3	Xinnan town	5	27°32′25.60″ N, 107°19′18.18″ E	XNC	XNU	XNL
4	Xima town	5	27°57′29.25″ N, 107°31′7.84″ E	XMC	XMU	XML
5	Yuquan town	5	27°52′26.22″ N, 107°29′34.00″ E	YQC	YQU	YQL
6	Mashan town	5	28°3′16.54″ N, 107°35′20.88″ E	MSC	MSU	MSL
7	Xihe town	5	28°9′19.95″ N, 107°32′30.31″ E	XHC	XHU	XHL
8	Yongxing town	5	27°52′30.94″ N, 107°35′26.49″ E	YXC	YXU	YXL
9	Gaotai town	5	27°37′13.25″ N, 107°23′21.92″ E	GTC	GTU	GTL
10	Chaole town	5	27°40′30.97″ N, 107°34′6.49″ E	CLC	CLU	CLL
11	Meijiang street	5	27°46′22.01″ N, 107°28′59.33″ E	MJC	MJU	MJL
12	Huangjiaba town	5	27°44′22.12″ N, E107°25′32.78″ E	HJC	HJU	HJL

**Table 2:** Standard curve formulation of each element.

Element	Wavelength/nm	Standard Curve Formulation	Correlation Coefficient/r	RSD/%
Cd	214.439	y = 12447.7579x + 0.4802	0.9908	1.55
Fe	238.204	y = 24370.3355x + 1019.2927	0.9983	0.37
La	333.749	y = 79738.8536x + 191.1089	0.9999	0.40
Mg	279.553	y = 540801.9881x + 7210.5008	0.9955	0.52
Mn	257.610	y = 168638.21418x + 1366.0338	0.9944	1.57
Ni	231.604	y = 2826.8109x + 44.7979	0.9999	0.53
Se	196.026	y = 535.1290x + 7.5406	0.9999	3.68
Pr	417.939	y = 25174.0633x + 26.5759	0.9998	2.05
Sm	359.259	y = 40242.5396x + 7.9324	0.9999	1.99
Zn	213.857	y = 25455.3631x + 148.4222	0.9999	0.35

**Table 3:** *I_geo_* Classification standard.

Index of Geoaccumulation	Grade	The Degree of Pollution
*I_geo_* < 0	Level 0	No pollution
0 ≤ *I_geo_* < 1	Level 1	No pollution to moderate pollution
1 ≤ *I_geo_* < 2	Level 2	Moderate pollution
2 ≤ *I_geo_* < 3	Level 3	Moderate to strong pollution
3 ≤ *I_geo_* < 4	Level 4	Strong pollution
4 ≤ *I_geo_* < 5	Level 5	Heavy pollution to extreme pollution
*I_geo_* ≥ 5	Level 6	Extreme pollution

**Table 4:** The contents of 10 elements in the topsoil and subsoil in 12 towns of Meitan (mg·kg^−1^).

10	Sample Information	Cd	Fe	La	Mg	Mn	Ni	Se	Pr	Sm	Zn
The topsoil	XMU	0.783 ± 0.007 ^g^	4260.5 ± 14.7 ^c^	1.579 ± 0.001 ^e^	52.61 ± 0.24 ^e^	0.16 ± 0.02 ^k^	4.015 ± 0.008 ^d^	5.35 ± 0.15 ^b^	0.9 ± 0.02 ^e^	1.12 ± 0.003 ^e^	1.62 ± 0.02 ^d^
	GTU	0.842 ± 0.002 ^c^	4416 ± 11.4 ^a^	1.603 ± 0.004 ^d^	48.93 ± 0.35 ^h^	0.20 ± 0.006 ^j^	3.953 ± 0.024 ^e^	5.87 ± 0.13 ^a^	0.9 ± 0.01 ^e^	1.141 ± 0.012 ^d^	1.83 ± 0.02 ^b^
	XHU	0.734 ± 0.008 ^h^	4013.5 ± 2.2 ^e^	1.893 ± 0.005 ^a^	52.09 ± 0.07 ^f^	0.586 ± 0.003 ^c^	3.456 ± 0.019 ^h^	5.03 ± 0.16 ^d^	0.99 ± 0.01 ^b^	1.363 ± 0.005 ^a^	1.635 ± 0.007 ^cd^
	FXU	0.856 ± 0.006 ^b^	4351.5 ± 11.8 ^b^	1.677 ± 0.001 ^c^	47.97 ± 0.12 ^i^	0.588 ± 0.001 ^c^	4.495 ± 0.012 ^c^	5.89 ± 0.12 ^a^	0.98 ± 0.01 ^c^	1.273 ± 0.002 ^b^	1.648 ± 0.005 ^c^
	HJU	0.824 ± 0.001 ^e^	4145.5 ± 6.5 ^d^	1.829 ± 0.005 ^b^	66.13 ± 0.06 ^c^	0.435 ± 0.001 ^e^	4.914 ± 0.015 ^a^	5.75 ± 0.14 ^a^	0.94 ± 0.005 ^d^	1.271 ± 0.006 ^b^	2.017 ± 0.007 ^a^
	MJU	0.729 ± 0.007 ^h^	3816 ± 7.2 ^g^	1.342 ± 0.005 ^h^	49.37 ± 0.22 ^g^	0.322 ± 0.004 ^h^	3.431 ± 0.032 ^i^	4.75 ± 0.06 ^e^	0.72 ± 0.02 ^gh^	1.013 ± 0.004 ^h^	1.24 ± 0.03 ^e^
	YQU	0.663 ± 0.005 ^j^	3560.5 ± 8.367 ^i^	1.068 ± 0.002 ^k^	43.06 ± 0.12 ^j^	0.246 ± 0.003 ^i^	3.061 ± 0.027 ^j^	4.17 ± 0.05 ^f^	0.63 ± 0.01 ^i^	0.864 ± 0.008 ^i^	1.06 ± 0.03 ^g^
	CLU	0.554 ± 0.005 ^k^	2896 ± 1.4^k^	1.358 ± 0.003 ^g^	191.62 ± 0.47 ^a^	2.228 ± 0.003 ^a^	3.519 ± 0.016 ^g^	3.54 ± 0.07 ^g^	4.37 ± 0.01 ^a^	0.54 ± 0.005 ^j^	1.005 ± 0.003 ^h^
	MSU	0.871 ± 0.006 ^a^	3875.5 ± 24.1 ^f^	1.385 ± 0.007 ^f^	69.18 ± 0.07 ^b^	0.423 ± 0.008 ^f^	4.834 ± 0.020 ^b^	5.74 ± 0.18 ^a^	0.732 ± 0.008 ^g^	1.094 ± 0.005 ^f^	1.62 ± 0.02 ^d^
	XNU	0.816 ± 0.01 ^f^	3589 ± 16.7 ^h^	1.166 ± 0.005 ^j^	49.61 ± 0.12 ^g^	0.727 ± 0.015 ^b^	4.492 ± 0.010 ^c^	5.18 ± 0.14 ^cd^	0.642 ± 0.003 ^i^	1.163 ± 0.002 ^c^	1.21 ± 0.02 ^f^
	YXU	0.832 ± 0.004 ^d^	3581.5 ± 2.9 ^h^	1.036 ± 0.007 ^l^	40.52 ± 0.1 ^k^	0.467 ± 0.011 ^d^	3.974 ± 0.019 ^e^	5.28 ± 0.17 ^bc^	0.703 ± 0.008 ^h^	1.061 ± 0.006 ^g^	1.07 ± 0.007 ^g^
	SLU	0.701 ± 0.001^i^	3524.5 ± 15.4 ^j^	1.310 ± 0.006 ^i^	61.93 ± 0.28 ^d^	0.398 ± 0.01 ^g^	3.752 ± 0.006 ^f^	4.3 ± 0.05 ^f^	0.754 ± 0.009 ^f^	1.162 ± 0.004 ^c^	0.86 ± 0.02 ^i^
	Mean value	0.767 ± 0.094 ^b^	3835.8 ± 436.3 ^a^	1.44 ± 0.28 ^b^	64.42 ± 40.99 ^b^	0.565 ± 0.55 ^b^	3.991 ± 0.589 ^b^	5.07 ± 0.75 ^b^	1.11 ± 1.04 ^b^	1.089 ± 0.216 ^b^	1.402 ± 0.371 ^b^
The subsoil	XML	0.859 ± 0.005 ^B^	4500.5 ± 5.7 ^A^	1.683 ± 0.002 ^A^	59.26 ± 0.27 ^C^	0.580 ± 0.012 ^C^	4.148 ± 0.009 ^G^	5.79 ± 0.16 ^AB^	0.943 ± 0.004 ^B^	1.133 ± 0.008 ^E^	1.966 ± 0.008 ^A^
	GTL	0.813 ± 0.008 ^E^	4279 ± 14.3 ^B^	1.554 ± 0.001 ^C^	51.63 ± 0.1 ^G^	0.542 ± 0.012 ^D^	4.046 ± 0.024 ^I^	4.29 ± 0.26 ^G^	0.799 ± 0.007 ^E^	1.086 ± 0.006 ^H^	1.687 ± 0.003 ^C^
	XHL	0.757 ± 0.008 ^G^	4053.5 ± 7.4 ^E^	1.599 ± 0.01 ^B^	53.21 ± 0.08 ^F^	0.487 ± 0.009 ^E^	3.924 ± 0.013 ^J^	5.13 ± 0.05 ^E^	0.825 ± 0.021 ^D^	1.158 ± 0.004 ^C^	1.52 ± 0.02 ^D^
	FXL	0.822 ± 0.005 ^D^	4161 ± 10.2 ^C^	1.529 ± 0.006 ^D^	54.51 ± 0.07 ^E^	0.456 ± 0.004 ^F^	4.077 ± 0.009 ^H^	5.74 ± 0.17 ^ABC^	0.891 ± 0.007 ^C^	1.117 ± 0.002 ^F^	1.91 ± 0.02 ^B^
	HJL	0.836 ± 0.003 ^C^	4066.5 ± 10.5 ^E^	1.56 ± 0.006 ^C^	63.55 ± 0.17 ^B^	0.375 ± 0.004 ^I^	4.796 ± 0.011 ^B^	5.58 ± 0.15 ^CD^	0.771 ± 0.012 ^F^	1.095 ± 0.002 ^G^	1.39 ± 0.03 ^F^
	MJL	0.857 ± 0.01 ^B^	4087.5 ± 4.7 ^D^	1.494 ± 0.004 ^F^	57.76 ± 0.05 ^D^	0.423 ± 0.002 ^G^	4.361 ± 0.025 ^C^	5.65 ± 0.09 ^BCD^	0.809 ± 0.01 ^DE^	1.142 ± 0.002 ^D^	1.44 ± 0.01 ^E^
	YQL	0.889 ± 0.007 ^A^	4082 ± 7.4 ^D^	1.501 ± 0.004 ^E^	51.47 ± 0.21 ^G^	0.363 ± 0.004 ^J^	4.311 ± 0.015 ^D^	5.88 ± 0.13 ^A^	0.948 ± 0.009 ^B^	1.19 ± 0.008 ^B^	0.98 ± 0.02 ^J^
	CLL	0.38 ± 0.004 ^H^	2062.5 ± 5.95 ^I^	1.357 ± 0.001 ^H^	234.71 ± 2.48 ^A^	1.108 ± 0.002 ^A^	3.769 ± 0.019 ^K^	2.12 ± 0.18 ^H^	14.08 ± 0.03 ^A^	0.456 ± 0.002 ^I^	1.052 ± 0.007 ^I^
	MSL	0.83 ± 0.009 ^CD^	3681 ± 11.8 ^F^	1.387 ± 0.003 ^G^	58.41 ± 0.11 ^CD^	0.450 ± 0.004 ^F^	4.214 ± 0.035 ^F^	5.48 ± 0.11 ^D^	0.767 ± 0.011 ^F^	1.201 ± 0.005 ^A^	1.303 ± 0.025 ^G^
	XNL	0.856 ± 0.004 ^B^	3694 ± 13.4 ^F^	1.249 ± 0.001 ^J^	53.22 ± 0.02 ^F^	0.410 ± 0.012 ^H^	4.246 ± 0.021 ^E^	5.57 ± 0.15 ^CD^	0.770 ± 0.005 ^F^	1.111 ± 0.003 ^F^	1.163 ± 0.014 ^H^
	YXL	0.852 ± 0.008 ^B^	3555 ± 25.2 ^H^	1.162 ± 0.007 ^K^	46.86 ± 0.14 ^H^	0.807 ± 0.013 ^B^	4.903 ± 0.016 ^A^	5.15 ± 0.17 ^E^	0.809 ± 0.006 ^DE^	1.208 ± 0.012 ^A^	0.958 ± 0.019 ^J^
	SLL	0.77 ± 0.003 ^F^	3647.5 ± 7.7 ^G^	1.332 ± 0.009 ^I^	62.66 ± 0.12 ^B^	0.408 ± 0.003 ^H^	3.941 ± 0.013 ^J^	4.77 ± 0.13 ^F^	0.758 ± 0.016 ^F^	1.207 ± 0.009 ^A^	0.354 ± 0.016 ^K^
	Mean value	0.79 ± 0.14 ^B^	3822.5 ± 623 ^A^	1.45 ± 0.15 ^B^	70.6 ± 51.91 ^B^	0.53 ± 0.22 ^B^	4.23 ± 0.34 ^B^	5.1 ± 1.04 ^B^	1.93 ± 3.83 ^B^	1.09 ± 0.21 ^B^	1.31 ± 0.45 ^B^

Note: There were significant differences in the same column data of the same soil layer (represented by different letters, *p* < 0.05).

**Table 5:** The contents of 10 elements in tea from 12 townships in Meitan (mg·kg^−1^, n = 5).

Sample Information	Cd	Fe	La	Mg	Mn	Ni	Se	Pr	Sm	Zn
SLC	0.012 ± 0.002 ^bc^	0.563 ± 0.018 ^k^	0.254 ± 0.002 ^a^	87.79 ± 0.39 ^i^	10.174 ± 0.053 ^a^	0.59 ± 0.02 ^f^	0.145 ± 0.047 ^cde^	0.266 ± 0.013 ^a^	0.006 ± 0.002 ^d^	1.165 ± 0.016 ^g^
FXC	0.006 ± 0.001 ^ef^	0.776 ± 0.004 ^j^	0.171 ± 0.003 ^c^	90.76 ± 0.22 ^h^	5.676 ± 0.055 ^i^	0.621 ± 0.044 ^ef^	0.093 ± 0.013 ^f^	0.137±0.005 ^d^	0.011 ± 0.004 ^ab^	1.12 ± 0.02 ^g^
XNC	0.005 ± 0.001 ^f^	1.19 ± 0.02 ^h^	0.166 ± 0.004 ^de^	109.07 ± 1.06 ^g^	6.135 ± 0.008 ^h^	0.678 ± 0.045 ^d^	0.259 ± 0.039 ^a^	0.121 ± 0.007 ^e^	0.007 ± 0.003 ^cd^	1.325 ± 0.005 ^f^
XMC	0.01 ± 0.004 ^cd^	1.754 ± 0.036 ^f^	0.162 ± 0.001 ^fg^	122.71 ± 0.89 ^d^	6.117 ± 0.081 ^h^	0.641 ± 0.029 ^de^	0.101 ± 0.024 ^f^	0.108 ± 0.003 ^f^	0.01 ± 0.002 ^abc^	1.386 ± 0.033 ^e^
YQC	0.005 ± 0.001 ^f^	2.02 ± 0.031 ^d^	0.146 ± 0.001 ^i^	140.94 ± 0.42 ^b^	7.074 ± 0.073 ^f^	0.895 ± 0.05 ^b^	0.116 ± 0.031 ^ef^	0.084 ± 0.012 ^g^	0.012 ± 0.003 ^a^	1.523 ± 0.106 ^d^
MSC	0.006 ± 0.003 ^ef^	2.485 ± 0.043 ^b^	0.157 ± 0.003 ^h^	142.64 ± 0.55 ^a^	9.051 ± 0.068 ^c^	0.984 ± 0.018 ^a^	0.171 ± 0.027 ^c^	0.089 ± 0.007 ^g^	0.007 ± 0.003 ^cd^	1.789 ± 0.04 ^a^
XHC	0.006 ± 0.001 ^f^	0.925 ± 0.047 ^i^	0.242 ± 0.003 ^b^	114.05 ± 0.95 ^f^	9.121 ± 0.09 ^c^	0.791 ± 0.03 ^c^	0.225 ± 0.034 ^ab^	0.212 ± 0.007 ^b^	0.008 ± 0.001 ^bcd^	1.276 ± 0.029 ^f^
YXC	0.013 ± 0.001 ^ab^	1.563 ± 0.026 ^g^	0.168 ± 0.002 ^cd^	128.99 ± 1.35 ^c^	6.858 ± 0.098 ^g^	0.756 ± 0.026 ^c^	0.122 ± 0.019 ^def^	0.123 ± 0.008 ^e^	0.012 ± 0.002 ^a^	0.319 ± 0.059 ^h^
GTC	0.007 ± 0.003 ^ef^	2.238 ± 0.024 ^c^	0.16 ± 0.003 ^g^	129.01 ± 0.931 ^c^	7.921 ± 0.046 ^e^	0.915 ± 0.021 ^b^	0.154 ± 0.016 ^cd^	0.093 ± 0.005 ^g^	0.009 ± 0.004 ^abc^	1.703 ± 0.035 ^b^
HJC	0.009 ± 0.001 ^de^	2.613 ± 0.081 ^a^	0.157±0.003 ^h^	141.51 ± 0.37 ^b^	9.876 ± 0.028 ^b^	0.924 ± 0.025 ^b^	0.121 ± 0.03 ^def^	0.107 ± 0.009 ^f^	0.011 ± 0.003 ^ab^	1.834 ± 0.012 ^a^
CLC	0.005 ± 0.001 ^f^	2.225 ± 0.034 ^c^	0.164 ± 0.002 ^ef^	121.79 ± 0.2 ^d^	3.715 ± 0.026 ^j^	1.01 ± 0.01 ^a^	0.128 ± 0.017 ^def^	0.12 ± 0.006 ^e^	0.012 ± 0.001 ^a^	1.606 ± 0.015 ^c^
MJC	0.015 ± 0.003 ^a^	1.892 ± 0.024 ^e^	0.166 ± 0.002 ^de^	117.9 ± 0.75 ^e^	8.229 ± 0.039 ^d^	0.593 ± 0.009 ^f^	0.218 ± 0.018 ^b^	0.149 ± 0.004 ^c^	0.009 ± 0.001 ^abc^	1.526 ± 0.007 ^d^

Note: There are significant differences in different Meitan tea areas for the same element (represented by different letters, *p* < 0.05).

## Data Availability

The datasets generated and analyzed during the current study are available from the corresponding author on reasonable request.
